# Probiotic Characterization of Lactic Acid Bacteria from Donkey Feces in China

**DOI:** 10.3390/ani15020207

**Published:** 2025-01-14

**Authors:** Yanqiu Wu, Shousong Yue, Jinhui Yu, Fei Bian, Gao Chen, Yan Zhang

**Affiliations:** 1 Institute of Crop Germplasm Resources, Shandong Academy of Agricultural Sciences, Jinan 250100, China; 2 Jinan Engineering Research Center of Conservation of Agricultural Microbial Resources and Biomanufacturing, Jinan 250100, China; 3 Jinan Key Laboratory of Conservation and Utilization of Agricultural Microbial Resources, Jinan 250100, China

**Keywords:** antimicrobial activity, probiotic characteristics, donkey husbandry, *Ligilactobacillus salivarius*

## Abstract

Probiotics are beneficial to humans and animals and often used for regulating immunity, intestinal microbiota balance, and animal growth performance. Donkey husbandry has boomed in China in recent years and there is an urgent need for probiotics effective for improving donkey health. However, studies on potential probiotic strains isolated from donkeys are scarce. This project aimed to screen LAB strains from donkey feces, detect their antimicrobial activity and evaluate their probiotic characteristics in vitro. Thirteen LAB isolates showed different degrees of antimicrobial activity against four indicator bacteria: three common pathogens (*Escherichia coli*, *Staphylococcus aureus*, and *Salmonella typhimurium*) and one pathogen restricted to equines (*Salmonella. abortus equi*), eight of which could inhibit all four pathogens. Seven isolates showed higher tolerance to low pH and bile salts, with >50% and >60% survival rates, respectively. Five of them had more than 50% survival rate to artificial gastric and intestinal fluids. Only three isolates possessed good properties, with >40% auto-aggregation, >40% hydrophobicity, and high co-aggregation with the indicator pathogens. An L9 isolate, identified as *Ligilactobacillus salivarius*, was sensitive to most antibiotics tested. Overall, these results indicate that the *L. salivarius* L9 isolate meets the requirements of the probiotics selection criteria in vitro and can potentially be developed as a probiotic for donkeys.

## Simple Summary

Probiotics are beneficial for human and animal health and have been used as dietary supplements for various purposes, such as enhancing immunity, improving growth performance, and modulating gut microbiota. Probiotics have host-specific characteristics that are better when obtained from the host. Donkey husbandry in China has been booming during recent years; however, there is a shortage of probiotics specific for donkeys. To develop probiotics for donkeys, this study explored the donkey-derived lactic acid bacteria (LAB) to inhibit common pathogens and those restricted to equines. Eight donkey-derived isolates exhibited antibacterial activity against four indicator pathogens tested. The isolates were selected based on their potential in vitro probiotic characteristics. *Ligilactobacillus salivarius* L9 strain isolated in this study had excellent probiotic properties, such as fast growth and acid production rates, tolerance to gastric and internal stress, higher hydrophobicity, auto-aggregation, co-aggregation activity with the four indicator pathogens, and absence of acquiring antibiotic resistance. L9 strain is a superior candidate probiotic and is expected to be applied in the donkey breeding industry after further validation in vivo experiments.

## 1. Introduction

In recent decades, overuse of antibiotics to enhance livestock growth has resulted in increased cases of antibiotic resistance, posing a major threat to human and animal health, as well as environmental safety [1]. Therefore, there is an urgent need for an effective antibiotic alternative that can result in relatively high animal yields and low mortality while maintaining environmental and consumer health. Among the various antibiotic substitutes, probiotics have been widely accepted due to their relatively safe sources and the benefits associated with feed supplementation, such as improved growth performance, meat quality, nutrient absorption, immune response, and inhibition of pathogen infection [2,3,4,5].

Probiotics are comprised of a large group of several types of bacteria. Among all probiotics groups, lactic acid bacteria (LAB) constitute the dominant group [6]. A large number of LAB strains have been isolated from fermented foods [7,8,9,10], raw milk [11,12], the gastrointestinal tract (GIT) [13,14], and the vagina [15,16]. Some of them have been used in food fermentation [17,18,19], feed additives [2,20,21], and therapeutic treatment [5,22,23,24].

LAB strains possess various potential probiotic functions in the host, such as enhancing immunity [25], reducing pathogen colonisation [26], and enhancing intestinal barrier function [27]. Therefore, LAB are used as an animal feed additive to promote animal growth, performance, and health. Supplementing *Lactobacillus lactis* in the diet of growing-phase pigs could increase their final body weight and average daily gain and improve meat quality [2]. Administration of LAB probiotics to rabbits could significantly improve their body weight [4]. Moreover, administration of *Pediococcus pentosaceus* to mice could provide protection against dextran sulfate sodium (DSS)-induced colitis by regulating intestinal flora and function, immunological profiles, and gut barrier function [28]. Similar results have been obtained in other animals, such as poultry [27,29,30], calves [26,31], and ostriches [32].

Potential probiotic strains from equus animals have also been isolated, such as *Lactobacillus pentosus*, *Lactobacillus. plantarum* isolated from horse feces [33,34]. Administration of LAB from healthy horses to mice could alleviate *Salmonella* infection and regulate intestinal flora [35].

However, probiotics have host-specific characteristics [36], so probiotic strains are best isolated from the hosts. Donkeys are herbivores with a monogastric digestive system. In recent years, donkey husbandry has risen due to the ever-increasing demand for donkey products in China. However, there are few studies on donkey-derived LAB [37,38]. The purpose of this study was to screen and identify LAB strains from healthy donkeys, evaluate their safety and potential probiotic characteristics, and lay the foundation for their application in donkey husbandry.

## 2. Materials and Methods

### 2.1. Isolation and Preliminary Identification

Healthy donkey feces were collected from scale donkey farms in Shandong Province, China, where no microbial feed additives or antibiotics were administered. After defecation, fresh feces were collected immediately in sterile fecal collectors and transferred to the laboratory in a cooler. Samples were 10-fold gradient diluted in 0.9% NaCl (*w*/*v*), and thereafter appropriate dilutions were spread onto De Man Rogosa Sharp (MRS) (Luqiao, Beijing, China) agar plates containing CaCO_3_ (10 g/L) and cycloheximide (Sigma–Aldrich, St. Louis, MO, USA) (100 mg/L). The plates were incubated at 37 °C anaerobically for 2–3 days. Suspected colonies with calcium-dissolving rings were selected to further Gram staining and catalase tests. Isolates possessing both gram-positive and catalase-negative attributes were sub-cultured for purification and then preserved at −80 °C in 20% glycerol.

### 2.2. Antimicrobial Activity

The antagonistic effect of selected LAB isolates was investigated with the Oxford cup method, according to a previous description [14], with slight modifications. LAB isolated fresh cultures in MRS were cultivated at 37 °C for 24 h at 1% inoculation and centrifuged (10,000 r/min, 5 min), and filter-sterilized using a 0.22 µm filter (Millipore, Watford, UK) to prepare cell-free culture supernatant (CFCS) filtered samples. *Escherichia coli* (ATCC 25922), *Staphylococcus aureus* (ATCC 25923), *Salmonella typhimurium* (ATCC 14028), and *Salmonella abortus equi* (CGMCC 18046), which causes abortions in donkeys, were used as indicator strains. A 100 μL indicator fresh culture (concentration of approximately 10^6^ CFU/mL) after incubation in LB medium at 37 °C overnight was spread evenly onto the LB agar plate. Oxford cups were pressed onto the LB plate and filled with 200 μL CFCS, or MRS medium without bacteria as the blank control, and subsequently pre-diffused for 4 h at 4 °C. Then, the plates were transferred to 37 °C and cultivated for 24 h, whereafter the inhibition zone diameters were determined with a vernier caliper (Deli, Ningbo, China).

### 2.3. Growth Curve and Acid Production Rate

Growth rate and acid production rate were tested according to a previous report [39], with slight changes. Fresh cultures of LAB isolates after two-round-subculture were added into 100 mL fresh MRS broth medium in an Erlenmeyer flask with a 1% inoculation level and cultured statically at 37 °C for 24 h. Then, 1.5 mL cultures were picked up to determine growth curve and pH value. Growth curve was measured via optical density (OD) at 600 nm using a spectrophotometer (Spectrum Instruments a PerkinElmer Company, Shanghai, China), and acid production rate was determined by measuring the pH value of the media with a pH meter (Shanghai Jingke Industrial Co., Ltd, Shanghai, China). All experiments were repeated three times.

### 2.4. Tolerance to Acid and Bile Salts

Evaluation of tolerance to different pH conditions was conducted as previously reported [40], with slight modifications. Fresh cultures of LAB isolates were anaerobically cultivated overnight. Then, 5 mL cultures were harvested (5000 r/min, 8 min), washed twice with phosphate-buffered saline (PBS, pH 7.4), and resuspended to approximately 10^8^ CFU/mL in MRS broth with various pH values (2.0, 3.0, and 4.0). Samples were picked up after 0 and 3 h and 10-fold gradient diluted, and appropriate dilutions were spread onto MRS agar plates. Viable colony counts were calculated after 48 h. The survival rate (SR) was calculated according to the following formula:

SR (%) = (N_1_/N_0_) × 100 (1)

where N_1_ and N_0_ are the viable colony count (log_10_ CFU/mL) of the selected LAB after incubation for 3 h and 0 h separately.

Assessment of tolerance to bile salts was conducted as previously reported [41], with some changes. Cell preparation was identical to that for tolerance to acid, but the cells were finally resuspended in MRS broth containing 0.1%, 0.2%, and 0.3% bovine bile salt (Sangon, Shanghai, China), and cultivated at 37 °C for 4 h. The SR was calculated as described above.

### 2.5. Tolerance to Artificial Gastric and Intestinal Fluids

Gastric and international fluid tolerance of LAB isolates was measured according to a previous report [42], with minor modifications. PBS containing 3 mg/mL pepsin (Sangon, Shanghai, China) was adjusted to pH 3.0 to prepare the artificial gastric fluid. PBS containing 1 mg/mL trypsin (Sangon, Shanghai, China) and 3 mg/mL bovine bile salt was adjusted to pH 8.0 to prepare the artificial intestinal fluid. Both solutions were filter-sterilized using a 0.22 µm filter. Cell preparation was the same as the method for tolerance to acid, but the cells were finally resuspended in artificial gastric fluid or intestinal fluid separately. The viable colony counts of cells in artificial gastric fluid were determined after cultivation for 0 or 2 h, while those in artificial intestinal fluid were measured after incubation for 0 or 4 h. The SR was calculated according to the above formula.

### 2.6. Cell Surface Characteristics

#### 2.6.1. Hydrophobicity

The hydrophobicity (H%) was tested as reported by Kang et al. [16], with slight changes. The samples of LAB cells were prepared according to the procedure for tolerance to acid, but harvested cells were finally resuspended in PBS (pH 7.4) to obtain the initial samples. The initial absorbance (A_0_) of the samples was measured at 600 nm. A 2 mL initial sample was mixed with the same volume of solvent (xylene, chloroform, and ethyl acetate), and vortexed for 5 min. After incubation for 5 h at room temperature (RT), a 100 µL sample of the upper supernatant was taken to measure the absorbance (A_1_) at 600 nm. The H% was calculated by the following formula:

H% = (A_0_ − A_1_)/A_0_ × 100 (2)

#### 2.6.2. Auto-Aggregation Assay

Auto-aggregation (Auto-A%) activity was detected according to the method previously reported [43], with some changes. Cell suspensions were prepared as for hydrophobicity, and initial absorbance (A_0_) was determined at 600 nm. A 4 mL cell suspension of each strain was incubated at RT for 5 h, and 100 μL of upper supernatant was carefully collected to determine the absorbance (A_1_) at 600 nm. Auto-A% was calculated according to the following formula:

Auto-A% = (A_0_ − A_1_)/A_0_ × 100 (3)

#### 2.6.3. Co-Aggregation Assay

The co-aggregation (Co-A%) was carried out as in the previous description [43], with some modifications. The pathogenic strains used in the co-aggregation test included *E. coli* ATCC 25922, *S. typhimurium* ATCC 14028, *S. aureus* ATCC 25923, and *S. abortus equi* CGMCC 18046. Cell suspensions of LAB isolates and pathogens were prepared as described above. A 2 mL cell suspension of each LAB isolate and a 2 mL of the cell suspension of each pathogenic strain were mixed and vortexed for 10 s. A cell suspension of each strain was used as control. The absorbance of the LAB isolates (A_LAB_) and pathogenic bacteria (A_pat_) in the control tubes and in the mixture (A_LAB+pat_) was measured after stationary incubation for 5 h at RT. Co-A% was calculated according to the following formula:

Co-A% = [(A_LAB_ + A_pat_)/2 − A_LAB+pat_]/[(A_LAB_ + A_pat_)/2)] × 100 (4)

### 2.7. Safety Assessment

#### 2.7.1. Hemolytic Activity

Nonhemolytic activity is the essential character of probiotics. Hemolytic reactions were evaluated using 5% sheep blood agar plate by the typical signs of hemolysis, including β-, α-, and γ-hemolysis, which shows clear, green, and no zones around colonies, respectively. γ-hemolysis with no zones surrounding colonies shows nonhemolytic activity.

#### 2.7.2. Antibiotic Susceptibility

Antibiotic susceptibility was measured via the disk diffusion method [16]. The following 12 antibiotic disks (abbreviation; concentration) (Sangon, Shanghai, China) were tested: tetracycline (TC; 30 μg), gentamicin (GM; 10 μg), penicillin (P; 10 μg), vancomycin (VA; 30 μg), clindamycin (CM; 2 μg), kanamycin (KAN; 30 μg), erythromycin (EM; 15 μg), cephradine (RAD; 30 μg), ciprofloxacin (CIP; 5 μg), ceftriaxone (CTR; 30 μg), chloramphenicol (CHL; 30 μg), and norfloxacin (NOR; 10 μg). LAB cultures (concentration of approximately 10^6^ CFU/mL) were spread onto MRS agar plates with sterile cotton swabs, and then the antibiotic papers were placed onto the plates, and incubated at 37 °C for 48 h. Inhibition zone diameters were determined with a vernier caliper. Susceptibility was determined as described by Charteris [44].

### 2.8. Molecular Identification

A 16S rRNA sequence analysis was used to confirm the taxonomic identification of selected LAB isolates. Genomic DNA of LAB isolates was extracted following the protocol of the DNA extract min-kit (Tiangen, Beijing, China). The DNA fragments were PCR-amplified with the universal primers, 27F (5′-AGAGTTTGATCCTGGCTCAG-3′) and 1492R (5′-TACGGYTACCTTGTTA CGACTT-3′), and sequenced at the Qingke Company (Qingdao, China). The obtained 16S rRNA sequences were compared with reference sequences in the GenBank database through the Basic Local Alignment Search Tool (BLAST) (https://blast.ncbi.nlm.nih.gov/Blast.cgi, accessed on 2 December 2024), and subsequently a phylogenetic tree was constructed using the neighbor-joining method.

### 2.9. Carbon Sources Utilization

To investigate the carbon utilization, six carbon sources, i.e., glucose, sucrose, lactose, galactose, maltose, and fructose, were selected to be added individually into 100 mL of no-carbon basal medium at a concentration of 20 g/L. Fresh culture was added into the medium containing different carbon sources at 1% inoculation amount and OD_600_ values were measured after 12 h.

### 2.10. Characteristic of Antimicrobial Substances

Antimicrobial substances of the selected LAB strains, including bacteriocins, hydrogen peroxide, and organic acids, were further tested using the Oxford cup method, and samples were prepared as previously reported [11], with modifications. Overnight MRS broth cultures of LAB strains were centrifuged at 10,000 rpm/min for 5 min, and supernatants were divided into five equal portions for the following treatments: untreated; boiled at 100 °C for 5 min; treated with 1 mg/mL proteinase K (Solarbio, Beijing, China) for 2 h; neutralized to pH 7.0 with 6 N NaOH; treated with 0.5 mg/mL catalase (Solarbio, Beijing, China) for 2 h after pH adjusted to 7.0. Subsequently, all samples were sterilized using 0.22 μm filters. The approach employed for assessing the antagonistic effect of the supernatant on the indicator bacteria was identical to that utilized for evaluating the antimicrobial activity of LAB (Section 2.2).

### 2.11. Stastical Analysis

The data for antimicrobial activity are shown as Mean ± Standard Deviation (SD). All data were analyzed using SPASS TM software version 26.0 and graphs were generated with GraphPad Prism 5.0. The phylogenetic tree was constructed using MEGA 5.1.

## 3. Results

### 3.1. Bacteria Isolation

More than 40 strains with obvious calcium-dissolving rings were isolated, and 13 potential LAB strains (L1–L13) based on their gram-positive, catalase-negative, and rod- or coccus-shaped cell morphology characteristics were preliminarily chosen for further analysis.

### 3.2. In Vitro Antimicrobial Activity

Antibacterial potential of the selected 13 LAB strains was investigated using the Oxford cup assay. Their inhibitory activity against pathogens is shown in Table 1. Twelve strains presented inhibitory activity against *E. coli*, *S. aureus*, and *S. typhimurium*, except strain L4, at varying degrees. Only eight strains (L1, L7, L8, L9, L10, L11, L12, and L13) showed broad-spectrum inhibition against all the indicator pathogens, including against *S. abortus equi*. Therefore, these eight strains were selected for further research. Among them, strains L9, L10, and L12 showed higher antimicrobial activity, with an inhibition zone of 18.1–20.3 mm against *E. coli*, 18.9–21.5 mm against *S. aureus*, 18.2–20.2 mm against *S. typhimurium*, and 16.9–19.7 mm against *S. abortion equi*, separately.

### 3.3. Analysis of Growth and Acid Production 

**Figure 1:**
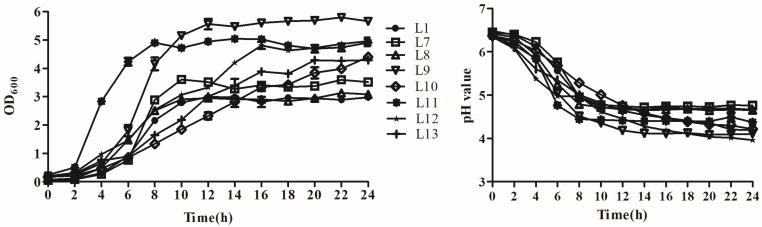
Growth and acid production rates of eight selected lactic acid bacteria strains isolated from donkey feces. Data are presented as the means of triplicate independent experiments. Error bars indicate standard deviations. OD_600nm_ = optical density measured at 600 nm.

Growth curves and acid production rate of the eight strains were also tested, and the results are shown in Figure 1. Among all tested strains, the L11 strain had the fastest growth but lower acid production rate. It first reached the growth stationary phase with the lowest pH level after 8 h, followed by strain L9, which had faster growth and acid production rates, reached the growth stationary phase after 12 h, and reached the lowest pH value of 4.12 after 14 h of incubation. Strain L12 shared a similar OD value with that of strain L11 after 16 h and reached a final pH of 3.96 after 24 h. Strain L10 grew slowly and did not reach the growth stationary phase until 24 h. Its pH level also declined slowly, arriving at a final pH of 4.22. The other strains exhibited relatively lower growth and acid production rates.

### 3.4. Analysis of Tolerance to the Gastrointestinal Environment 

#### 3.4.1. Acid Tolerance Analysis

**Figure 2:**
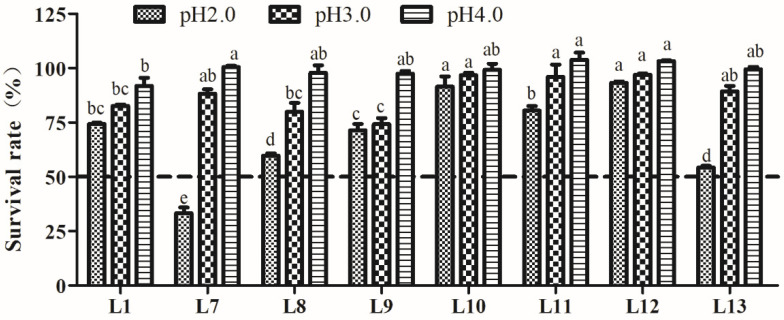
Tolerance of lactic acid bacteria strains isolated from donkey feces to pH 2.0, 3.0, and 4.0. Data show the means of triplicate independent experiments, and error bars represent standard deviations. The same letter indicated at the same pH value represents non-significant differences (*p* > 0.05) between isolates, whereas different letters represent significant differences (*p* < 0.05).

The tolerance to acid of the eight LAB strains is shown in Figure 2. The results indicated that all tested strains possessed a certain degree of tolerance to different acidities, which decreased following the pH decline. Little effect on SR was found after pH 3.0 and pH 4.0 treatment. However, acidity at pH 2.0 had a greater impact on the survival of LAB strains. Strain L12 exhibited a significantly higher tolerance to all three pH levels (*p* < 0.05). Strains L7, L8, and L13 showed a rapid decrease in SR at pH 2.0, especially strain L7, which exhibited a SR of only 26.6%, whereas the other seven strains showed SRs above 50%. Therefore, the remaining seven strains, excluding L7, were used for further experiments.

#### 3.4.2. Bile Salt Tolerance Analysis

**Figure 3:**
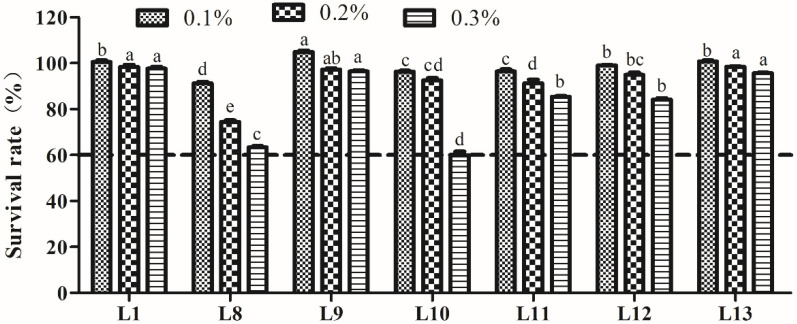
Tolerance of lactic acid bacteria strains isolated from donkey feces to 0.1, 0.2, and 0.3% bile salt concentrations. Data are shown as the means of triplicate independent experiments. Error bars represent standard deviations. The same letter at the same bile salt concentration represents non-significant differences (*p* > 0.05) between isolates, whereas different letters represent significant differences (*p* < 0.05).

The bile salt tolerance of LAB is shown in Figure 3. The SR of the seven strains decreased following increasing concentrations (0.1–0.3%) of bile salts. All strains possessed higher tolerance to different concentrations of bile salts, with an SR > 60.0%, even at a bile salt concentration of 0.3%. In particular, strains L1, L9, and L13 presented significantly higher tolerance to 0.3% bile salts (*p* < 0.05).

#### 3.4.3. Artificial Gastric and Intestinal Fluids Tolerance Analysis

**Figure 4:**
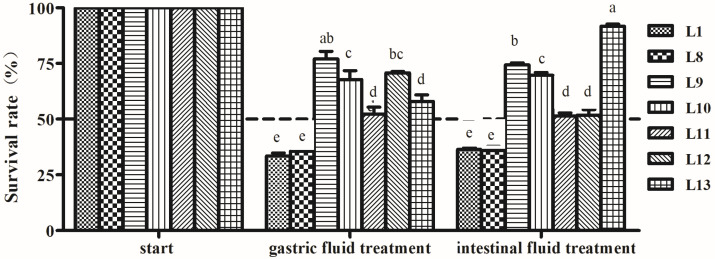
Tolerance of lactic acid bacteria strains isolated from donkey feces to artificial gastric and intestinal fluids. Data are shown as the means of triplicate independent experiments. Error bars represent standard deviations. The same letter in the same treatment represents non-significant differences (*p* > 0.05) between isolates, whereas different letters represent significant differences (*p* < 0.05).

The tolerance results of seven LAB isolates to gastric and intestinal fluids are shown in Figure 4. The survival rates of the strains were influenced by the treatment of gastric juice and intestinal juice, especially for L1 and L8 strains, exhibiting 33.5 and 35.6% SR for gastric juice and 36.4 and 35.9% SR for internal juice, respectively. The other strains showed high tolerance to gastric and intestinal fluids with SR > 50%. L13 strain showed that the SR of intestinal juice was the highest (91.7%), while the SR of gastric juice was lower (57.9%). Both L9 and L10 strains presented relative higher tolerance to gastric fluid (67.8 and 69.7% SR) and intestinal fluid (67.8 and 69.7% SR) (*p* < 0.05).

### 3.5. Analysis ofCell Surface Characteristics

#### 3.5.1. Auto-Aggregation and Co-Aggregation Assay

**Figure 5:**
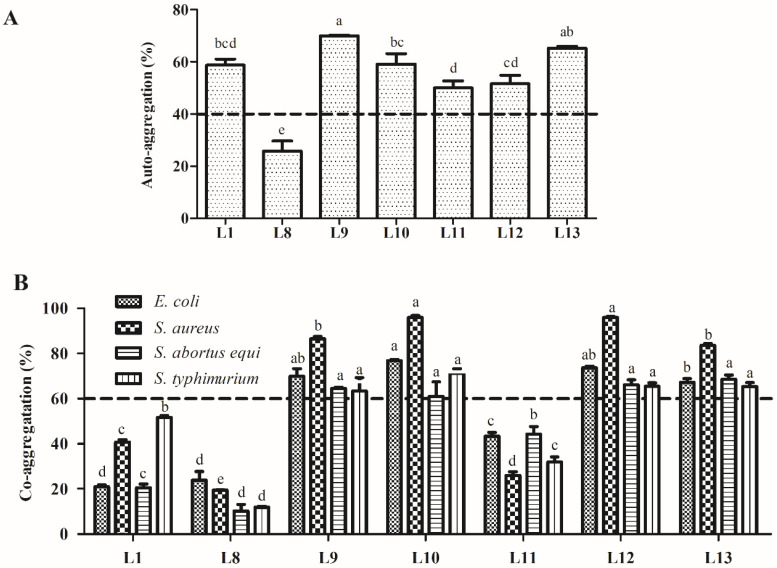
Auto-aggregation (**A**) and co-aggregation activities (**B**) of the lactic acid bacteria strains isolated from donkey feces. Data are shown as the means of triplicate independent experiments. Error bars indicate standard deviations. The same letter represents non-significant differences (*p* > 0.05) and different letters represent significant differences (*p* < 0.05) between isolates that auto-aggregated and co-aggregated with the four indicator pathogens, respectively.

The auto-aggregation and co-aggregation abilities of the seven tested LAB strains are presented in Figure 5. The LAB strains exhibited different auto-aggregation rates after incubation for 5 h ranging from 50.1% to 70.0%, except for strain L8, which had the lowest auto-aggregation rate (25.7%). Among all tested strains, strain L9 had a significantly higher auto-aggregation rate (70.0%, *p* < 0.05). Regarding co-aggregation, strains L9, L10, L12, and L13 displayed significantly higher co-aggregation rates with the four indicator pathogens, ranging from 61.0% to 96.1% (*p* < 0.05).

#### 3.5.2. Hydrophobicity Analysis

**Figure 6:**
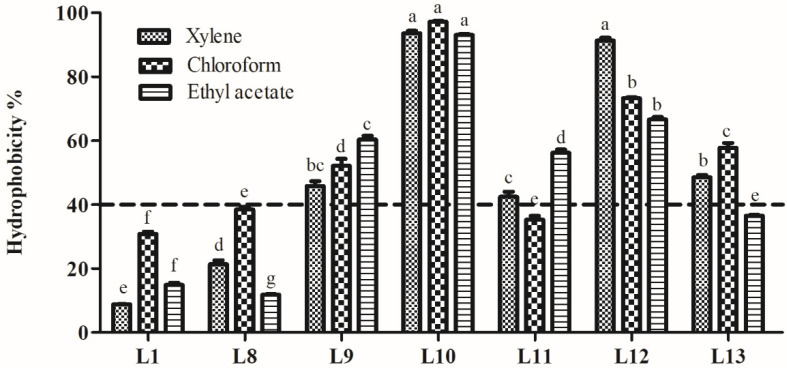
Hydrophobicity of lactic acid bacteria strains isolated from donkey feces. Data are shown as the means of triplicate independent experiments. Error bars represent standard deviations. The same letter in the same hydrocarbon group represents non-significant differences (*p* > 0.05) between isolates, whereas different letters represent significant differences (*p* < 0.05).

Hydrophobicity of the seven strains is shown in Figure 6. The hydrophobicity in xylene, chloroform, and ethyl acetate ranged from 8.8% to 93.5%, 30.8% to 97.2%, and 11.8% to 93.0%, respectively. Among the isolates, strain L10 showed significantly higher hydrophobicity in xylene, chloroform, and ethyl acetate (93.5%, 97.2%, and 93.0%, respectively; *p* < 0.05). Strains L12 and L9 exhibited more than 40% hydrophobicity in three hydrocarbons. Strains L11 and L13 showed hydrophobicity values above 40% for only two of the hydrocarbons tested. Based on the probiotic properties of the isolates tested in this research, strains L9, L10, and L12 were selected for further analysis.

### 3.6. Analysis of Safety 

To determine the safety of three LAB strains, hemolytic activity and antibiotic susceptibility were measured. The results are shown in Table 2. None of the three isolates exhibited hemolytic activity.

The antibiotic susceptibility results for 12 common antibiotics indicated that three isolates were resistant to two antibiotics (gentamicin and kanamycin) and sensitive to five (penicillin, chloramphenicol, erythromycin, ceftriaxone, and clindamycin). Of the 12 antibiotics, the L9 isolate was resistant to only three antibiotics (3/12, 25%) and sensitive to the rest (8/12, 75%). However, strains L10 and L12 were resistant to six antibiotics (6/12, 50%). The L9 strain, with the lowest antibiotic resistance and without acquiring antibiotic resistance, to including tetracycline, erythromycin and penicillin, was selected for further measurement.

### 3.7. Molecular Identification via 16S rRNA Sequencing

**Figure 7:**
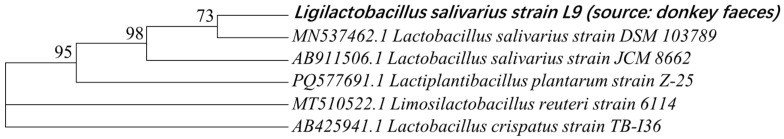
Phylogenetic tree based on the 16S rRNA gene sequence data of the selected L9 strain isolated from healthy donkey feces. The phylogenetic tree was constructed using the neighbor-joining method with 1000 bootstrap using MEGA 5.1.

Based on the potential probiotic characteristics measured for the isolates, the L9 strain was selected for further identification via 16S rRNA sequencing and BLAST alignment using the National Center of Biotechnology Information (NCBI) database. The L9 isolate was found to be highly consistent with *Ligilactobacillus salivarius* OR430873.1 (100% similarity) [Basonym: *Lactobacillus salivarius*] [45]. The phylogenetic tree based on the 16S rRNA sequences of the L9 isolate is shown in Figure 7.

### 3.8. Analysis of Carbon Source Utilization

**Figure 8:**
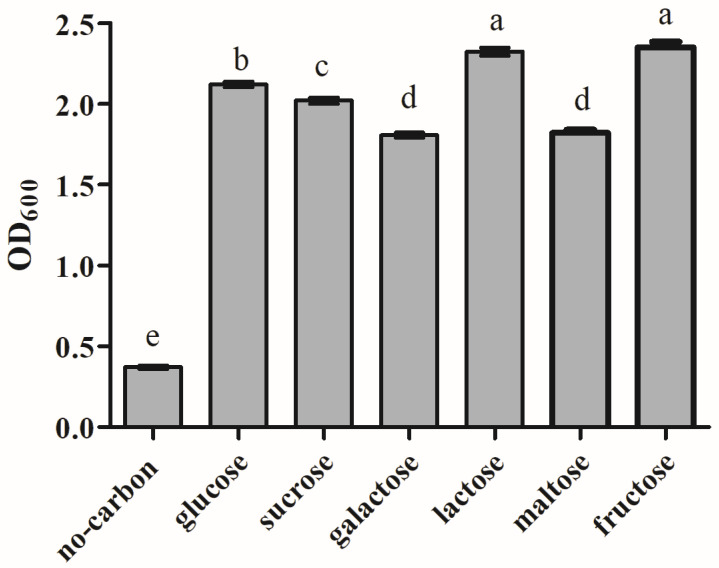
Effect on L9 isolate under different carbon source conditions. Data are shown as the means of triplicate independent experiments. Error bars represent standard deviations. The same letter represents non-significant differences (*p* > 0.05) between carbon sources, whereas different letters represent significant differences (*p* < 0.05).

The effect of carbon sources on the growth of L9 isolate is shown in Figure 8. The results indicated that all six carbon sources could support the growth of strain L9. Among them, lactose and fructose are the best carbon sources for strain L9, followed by glucose and sucrose.

### 3.9. Characteristics of Antimicrobial Substances from Lactic Acid Bacteria

To identify the antimicrobial substances produced by the LAB, which mainly included organic acids, hydrogen peroxide, and bacteriocins, treatments were carried out as shown in Table 3. The results showed that untreated and heat-treated CFCS of *L. salivarius* L9 strain possessed wide antagonistic activity against all four indicator pathogens, suggesting that antimicrobial substance(s) secreted by the L9 strain may not be heat sensitive. Furthermore, catalase-treated CFCS of the L9 strain had no effect on antagonistic activity against the four indicator pathogens, suggesting that antimicrobial activity was not attributed to hydrogen peroxide. Nevertheless, the neutralized CFCS of the L9 strain completely lost antimicrobial activity against the four indicator pathogens, indicating that the organic acids secreted by *L. salivarius* L9 were likely to be primarily responsible for its antimicrobial activity.

## 4. Discussion

Over the years, LAB have been screened for potential probiotics from different sources, including raw milk, fermented foods, and human and animal GITs. However, few studies on probiotics isolated from donkeys have been carried out. So far, only LAB strains from donkey milk have been isolated [38]. In order to obtain more probiotics for use in the donkey breeding industry, LAB strains were isolated from donkey feces and evaluated for their potential probiotic properties.

Antimicrobial activity is a key property of probiotic strains. In this study, the tested LAB strains exhibited different degrees of antagonistic effects on four indicator pathogens. *E. coli*, *S. aureus*, and *S. typhimurium* are common pathogens that cause diarrhea, sepsis, and severe infections. All LAB strains showed broader inhibition zones against *E. coli*, followed by *S. aureus* and *S. typhimurium*. Minimum inhibitory effect was observed against *S. abortus equi*, which is a pathogen restricted to equines [46] and the most common etiological agent resulted in abortion in mares, septicemia or polyarthritis in foals, and orchitis in stallions [47]. Eight LAB strains with antimicrobial activity against all four pathogens were selected for subsequent evaluation.

Our findings regarding antagonistic effects on both gram-positive and gram-negative bacteria are consistent with earlier studies [1,11]. The observed antagonistic activity of LAB strains showed no relationship with the gram classification of the pathogens, aligning with de Almeida Junior’s report [48].

Tolerance to acid and bile salts is an essential characteristic for potential probiotic strains, which allows them to survive and pass through the GIT. In this study, the tested LAB strains demonstrated robust growth at pH 4.0 and pH 3.0. Even at pH 2.0, which is usually used as an extreme GIT condition in vitro [49], they still showed an SR above 50% except for L7 strain. In terms of bile salt tolerance, seven strains exhibited high SRs, even at a 0.3% bile salt concentration. Previous research has also indicated that LAB strains from various sources possessed the ability to tolerate bile salts [50]. The high tolerance of LAB strains to acidity and bile salts in this study may be attributed to their origin from donkey feces, as they have already adapted to acid and bile salt stress in the GIT. However, subsequent treatments with gastric and internal fluids caused severe damage to some of the isolates. In particular, L1 and L8 isolates showed <50% SRs for both fluids. This suggests that the enzymes in GIT affected the survival of LAB strains, which was identical with the findings of the previous report [11].

Evaluations of auto- and co-aggregation are necessary for selecting effective probiotic strains. Auto-aggregation is the self-interaction within the same microbial strain, while co-aggregation is the aggregation between different microbial strains. These traits support potential probiotic adhesion to host GIT epithelial cells [51] and prevent pathogens from colonisation [52]. Auto-aggregation is strongly related to adhesion [8] and aids in biofilm formation, which protects the host from pathogen invasion [53]. In this study, six strains exhibited higher levels of auto-aggregation, ranging from 50.0% to 70.0%. The co-aggregation of probiotic strains with pathogens suggests their capacity to attach to them in vivo and create a microenvironment in which their antimicrobial metabolites can antagonise pathogenic bacteria [54]. In this study, four strains (L9, L10, L12, and L13) possessed higher levels of co-aggregation with the four tested indicator bacteria, ranging from 63.3% to 96.1%. Auto- and co-aggregation abilities of LAB show quite different results in the literature; Reuben et al. (2020) reported that LAB strains from cow and goat milk possessed higher co-aggregation (58.93–96.78%) and lower co-aggregation (5.96–62.64%), especially two strains from goat milk identified as *P. pentosaceus*, which exhibited similar auto-aggregation (41.5 and 45.5%, respectively) and a vastly different co-aggregation (5.96–33.19% and 30.16–62.64%, respectively) [11]. In comparison, the present study revealed that six strains exhibited higher auto-aggregation, but only four of them showed higher co-aggregation. The discrepancies in the auto- and co-aggregation results may be attributed to strain-specific characteristics and the source of the LAB strains. The cell-surface hydrophobicity of microbes is closely related to their adhesion to epithelial cells. Hydrophobicity with a minimum value of 40% is an essential prerequisite for probiotic strains [55]. Strains with higher cell-surface hydrophobicity possess a greater ability to adhere to intestinal mucosal cells [7]. In this study, three LAB strains (L9, L11, and L12) exhibited higher hydrophobicity, above 40% for the three solvents tested, showing values of 45.8–60.4%, 93.0–97.2%, and 66.7–91.3% in xylene, chloroform, and ethyl acetate, respectively. The relatively higher hydrophobicity measured in our study compared to that in others may be due to the different incubation times, which can affect hydrophobicity.

Nonhemolytic activity is a requirement and safety condition for probiotic strains. All strains isolated in our study exhibited the same nonhemolytic activity as that of probiotic strains isolated in previous studies [7,15,53].

Antibiotic susceptibility must be evaluated for all potential probiotic strains to avoid horizontal transfer of antibiotic resistance genes, which are harmful to the host. Major antibiotic genes cannot be transferred horizontally to recipient bacteria [56]. However, resistance to erythromycin, penicillin, or tetracycline is generally considered to be acquired by lactobacilli. Therefore, lactobacilli with resistance to these antibiotics are recognised as a threat to public security and rejected for application as potential probiotics [53]. Gentamycine and vancomycin resistance was considered to be intrinsic. Gentamycine resistant was due to lack of cytochrome-mediated electron transport [57] and vancomycin resistance was the result of cell wall structure [58]. In the present study, the L9 isolate, which possessed better probiotic properties, was sensitive to most of the antibiotics (9/12, 75.0%) tested and did not acquire antibiotic resistance.

Compared with that of biochemical identification, molecular identification via 16S rRNA sequencing is more rapid and precise. Based on 16S rRNA sequencing, the L9 strain was identified as *L. salivarius*, which is known to modulate innate immune responses, improve protection against intestinal viral–bacterial superinfection [59], and prevent antibiotic-associated diarrhoea [60].

The antimicrobial substances secreted by *L. salivarius* L9 were tested. These results suggest that the antimicrobial activity of *L. salivarius* L9 is likely attributable to the organic acids produced. Similarly, Reuben et al. in previous reports also demonstrated that acid production of some LAB strains from raw milk and poultry GIT was the major inhibitor of pathogens [11,14], but these are still good candidates for probiotics, due to their superior properties.

So far, some researches on donkey LAB have been reported, but mainly focused on its biotype or microbiota [37,61]. Some strains have already been isolated from donkey milk or fermented donkey milk [61,62], including isolates of *Lactobacillus mucosae*, *L. casei*, *L. paracasei*, *L. pentosus*, *L. mesenteroides*, *Lactiplantibacillus plantarum*, *Enterococcus* sp., *Leuconostoc lactis*. All these strains exhibited good probiotic properties, but this is the first time that LAB strains from donkey feces and a new specie, *L. salivarius*, derived from donkey were isolated. In our study, *L. salivarius* L9 possessed excellent potential probiotic properties. In particular, L9 strain exhibited a strong inhibitory effect on *S. abortus equi*, which causes serious economic losses to farmers. This is the first study to control *S. abortus equi*. with LAB. Our results indicate that *L. salivarius* L9 has possible applications in the donkey breeding industry. Further research on the feeding effect of *L. salivarius* in donkeys and the mechanisms involved require further investigation to obtain a highly effective probiotic additive for donkeys.

## 5. Conclusions

We conclude that *L. salivarius* strain L9 has probiotic potential in vitro, and antagonistic activity against common pathogens, including the donkey-specific *S. abortus equi*. This strain may be used as a feed additive in donkey husbandry to prevent diseases after further in vivo evaluation in donkeys.

## Figures and Tables

**Table 1:** Antimicrobial activity of the lactic acid bacteria isolated from donkey feces.

Isolates	Antimicrobial Activity			
	*E. coli* ATCC 25922	*S. aureus* ATCC 25923	*S. typhimurium* ATCC 14028	*S. abortus equi* CGMCC 18046
L1	16.1 ± 0.1	16.5 ± 0.5	15.7 ± 0.1	11.2 ± 0.3
L2	16.4 ± 0.2	15.4 ± 0.2	16.8 ± 0.2	-
L3	16.1 ± 0.1	14.1 ± 0.2	11.9 ± 0.1	-
L4	15.8 ± 0.3	-	11.0 ± 0.0	-
L5	16.0 ± 0.2	15.7 ± 0.3	14.6 ± 0.2	-
L6	17.2 ± 0.2	13.4 ± 0.2	15.0 ± 0.1	-
L7	16.7 ± 0.1	13.0 ± 0.1	16.5 ± 0.6	11.2 ± 0.3
L8	15.3 ± 0.3	13.3 ± 0.3	13.9 ± 0.1	13.4 ± 0.3
L9	18.1 ± 0.3	21.5 ± 0.2	20.2 ± 0.1	19.7 ± 0.5
L10	20.3 ± 0.8	21.1 ± 0.5	19.5 ± 0.1	19.4 ± 0.1
L11	16.7 ± 0.3	13.9 ± 0.1	13.8 ± 0.3	13.5 ± 0.3
L12	19.5 ± 0.1	18.9 ± 0.5	18.2 ± 0.2	16.9 ± 0.5
L13	17.1 ± 0.1	18.2 ± 0.3	14.6 ± 0.1	16.2 ± 0.1

-, diameter of the zone of inhibition ≤ 10 mm. Values indicate the diameter of the inhibitory zone (unit: mm). Data are shown as the mean of triplicate independent experiments ± standard deviations.

**Table 2:** Hemolytic activity and antibiotic sensitivity of selected lactic acid bacteria isolates.

Strains	Hemolytic	Antibiotic Sensitivity											
		TC	GM	P	VA	CC	K	E	CRD	CIP	CRO	C	NOR
L9	No	S	R	S	R	S	R	S	S	S	S	S	MS
L10	No	R	R	S	S	S	R	S	R	R	S	S	R
L12	No	R	R	MS	S	S	R	S	R	R	S	S	R

Antibiotic sensitivity: tetracycline (TC), gentamicin (GM), penicillin (P), vancomycin (VA), clindamycin (CC), kanamycin (K), erythromycin (E), cephradine (CRD), ciprofloxacin (CIP), ceftriaxone (CRO), chloramphenicol (C), norfloxacin (NOR). R, resistant; S, susceptible; MS, moderately susceptible.

**Table 3:** Antimicrobial activity of supernatants with different treatments against indicator bacteria.

Lactic Acid Bacteria Strain	Treatment	Zone of Inhibition (mm)			
		*E. coli*	*S. aureus*	*S. typhimurium*	*S. abortus equi*
	Untreated	+++	+++	+++	++
	Heat-treated	+++	+++	+++	++
L9	Neutralized	-	-	-	-
	Catalase-treated	+++	+++	+++	++

Symbols show inhibition zones (mm); -, no inhibition (<10 mm); ++, good (15–19 mm); +++, strong (>19 mm). Data represent measurements of three independent experiments.

## Data Availability

The data presented in this study are available by sending email to the corresponding author.
